# Exploring the psychological morbidity of waiting for sinus surgery using a mixed methods approach

**DOI:** 10.1186/s40463-016-0149-z

**Published:** 2016-06-07

**Authors:** Gordon Fung-Zak Tsang, Carmen L. McKnight, Laura Minhui Kim, John M. Lee

**Affiliations:** Department of Otolaryngology-Head & Neck Surgery, University of Toronto, Toronto, ON Canada; Department of Otolaryngology-Head & Neck Surgery, St. Michael’s Hospital, Toronto, ON Canada; Faculty of Medicine, University of Toronto, Toronto, ON Canada

**Keywords:** Rhinology, Chronic rhinosinusitis, Anxiety, Depression, Mixed methods, Qualitative, Survey, HADS, WPAI, Waitlist, Endoscopic sinus surgery

## Abstract

**Background:**

Patients with chronic rhinosinusitis (CRS) often have to endure significant wait times for endoscopic sinus surgery (ESS). The pyschiatric impact of placement on a waitlist for ESS has not been explored.

**Methods:**

Questionnaires measuring CRS symptom severity and health-related anxiety and stress (SNOT-22, HADS, WPAI-GH) were sent to patients diagnosed with CRS and currently on a waitlist for ESS. Fifteen representative waitlisted patients participated in one-on-one semi-structured interviews discussing their experience with their wait for ESS. A deductive thematic analysis was used to interpret the interview data using a quantitative driven mixed methods analysis.

**Results:**

Participants waiting for ESS reported worsening clinical symptomatology during their waiting period. Participants reported waitlist and CRS impact on both work and social aspects of their lives. The HADS scale showed no overall significant level of depression or anxiety in the HADS screening questionnaire. The qualitative data describe the effects of the symptom burden of CRS.

**Conclusions:**

Patients waitlisted for ESS did not demonstrate any significant level of psychiatric distress, however variability exists. The qualitative arm of this study elucidates how patients cope with their wait.

## Background

Chronic rhinosinusitis (CRS) with or without nasal polyposis is an inflammatory condition involving the mucosa of the nose and paranasal sinuses affecting approximately 5–15 % of the Canadian population [1]. Although initial treatment is generally non-surgical, surgical intervention may be required and efficacious in patients who are refractory to maximal medical treatment in terms of symptom relief [1]. Due to persisting funding issues for elective surgeries in our health care system, the wait time for surgery for CRS is often lengthy, averaging 164 days in the province of Ontario [2]. There is significant institutional variability and in Toronto ranges between 93 and 413 days (at the time of the submission of this study) [2]. Patients are often markedly symptomatic at the time of referral for surgery with previously demonstrated progression of Sinonasal Outcome Test 22 (SNOT-22) scores during their wait [3]. Thus, long wait times may significantly affect their psychological well-being and may lead to significant patient distress [4–13].

The current study explores the patient experience while waiting for ESS and the effect that wait times have on psychological morbidity and burden. A mixed methods approach was designed for this cross-sectional study. Questionnaires used include the SNOT-22, the Hospital Anxiety and Depression Scale (HADS), and the Work Productivity and Activity Impairment General Health (WPAI-GH) questionnaire. A qualitative component was used to help interpret and inform findings from the questionnaires and identify major contributors to psychological stressors.

## Methods

The study protocol was reviewed and approved by the central research ethics board (St. Michael’s Hospital, Toronto, ON) prior to starting data collection and analysis.

### Patient population and data collection

Data was accrued from a cross-section through a cohort of patients currently on a single surgeon’s (JL) waiting list for ESS for CRS during in 2014. The surgeon has a subspecialty focus in rhinology and anterior skull base surgery in a tertiary care hospital and there are approximately equal numbers of patients seen for primary and revision ESS for CRS. All patients were residents of Ontario, Canada. Inclusion criteria for participating patients on the waitlist include: documented diagnosis of CRS, patients underwent a trial of maximal medical therapy as outlined in current guidelines [1], ≥18 years of age, completed a consent to ESS for treatment of CRS (some consents included septoplasty as an adjunct procedure for obtaining adequate surgical access or when deemed required based on clinical symptoms), adequate fluency in English to provide informed consent and complete questionnaires, on waitlist for longer than 2 weeks. Exclusion criteria includes: patients who were pregnant or have been diagnosed with any of cystic fibrosis, immotile cilia syndrome, immunodeficiency syndrome, severe ongoing depression or drug addiction, or with medical reasons for delaying surgery. Additionally, we excluded patients who were urgently scheduled for ESS because of impending complications related to their illness (i.e. mucocele formation, significant skull base/orbital involvement). A chart review was performed on wait-listed patients producing a cohort of 68 patients who met study criteria (out of a total of 165 patients). An information sheet was mailed to all eligible participants inviting them to partake in a series of three questionnaires (SNOT-22, WPAI-GH, HADS).

During the chart review process, a sample of patients were purposefully chosen to provide a rich and meaningful representation of the experience of waiting for ESS. This sampling method is in keeping with qualitative studies [14]. The selected patients were then contacted by phone and asked to complete a series of three questionnaires and participate in a recorded, anonymous, one-on-one interview with the option of completing an in-person interview. The interview consisted of a verbal consent process followed by questions and were recorded on a digital recorder. The surgeon (JL) did not perform any of the interviews so as to minimize the risk of bias and also to allow participants to freely express their thoughts and feelings.

**Fig. 1:**
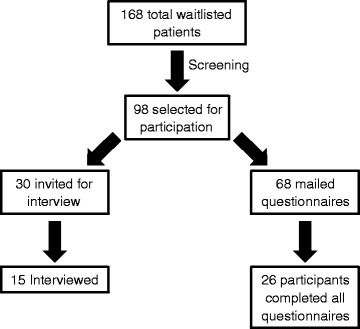
Flow diagram of study participants

Participants in the quantitative study portion were asked to participate by completing three questionnaires only. A total of 26 participants completed the questionnaires in full. All questionnaires were completed as either a hard copy or online. Participants choosing to complete the questionnaires online were emailed a unique identification code to input into a secured online survey website administered by Fluid Surveys (http://fluidsurveys.com/). The above selection process is summarized in Fig. 1.

### Demographic data

A chart review was performed for participating patients to ensure inclusion/exclusion criteria and to gather clinical and demographic data. A current “wait-time” was determined by calculating the number of days between the day their surgical consent was completed from the completion day of their questionnaires.

### Questionnaires

The SNOT-22 is a validated clinical tool used to objectively describe patient symptom from CRS burden and track clinical outcomes. It was used to help in the characterization of the patient population currently under study. The HADS tool is a 14 item self-implemented questionnaire that is divided into two sections, each designed to screen for states of anxiety and depression in the setting of a hospital medical outpatient clinic [15]. Each item is scored on a 4-point ordinal scale (range 0 to 3 with a higher score representing greater symptom severity) with a maximum total score of 21 in each depression and anxiety scales. The WPAI-GH questionnaire measures the impact of the patient’s condition on both their work and non-work activities over a seven day recall period prior to its completion [16]. This general health questionnaire was chosen specifically for two reasons. Firstly, a specific version for CRS has not been validated. Secondly, CRS is often comorbid with other airway disorders (i.e., asthma) or upper airway infections and the disease processes can often interact. The questionnaire consists of six questions regarding the current state of employment, hours missed due to health problems, hours missed for other reasons, hours actually worked, and degree that health affected productivity in regular unpaid activities [17]. Four main outcomes are generated from the WPAI-GH and expressed in percentages: 1) percent work time missed due to health for those who were currently employed; 2) percent impairment while working due to health for those who were currently employed and actually worked in the past seven days; 3) percent overall work impairment due to health for those who were currently employed; and 4) percent activity impairment due to health for all respondents [17].

### Patient interviews

**Fig. 2:**
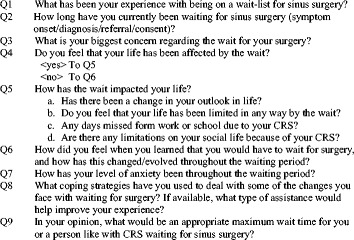
Interview Guide

The interviews were all performed in a one-on-one format. A single interviewer performed all the interviews (GT) in a semi-structured format. An interview guide was developed by the authors and used during the interviews to extract important themes (Fig. 2). Follow up questions were asked by the interviewer to clarify or develop thoughts and ideas from patients as needed. Exploration or other themes took place only if the interviewee introduced them. No time limit was set for the interviews, but the average interview was approximately 15 min long. The recorded interviews were transcribed and coded by the interviewer (GT) and subsequently proofread for accuracy by a second reviewer independently. The interviews were analyzed by the two aforementioned reviewers using a deductive thematic analysis approach. Using this approach, the goal was to extract data to help explain the quantitative results from the surveys. For the purposes of the qualitative study, an a priori target minimum of 10 participant interviews was established. Given the research question, no more than 10 interviews were seen to be needed especially since a broad range of waitlisted participants were selected. A total of 15 interviews were completed to ensure information saturation. After the interviews, the participants were also requested to complete a set of questionnaires.

### Data analysis

Standard summary statistics were calculated for all survey results. All statistical tests were 2-sided with *P* values less than 0.05 deemed significant. Statistical analysis (linear regression) was performed on SPSS version 23.0.

## Results

### Demographics

A total of 26 patients completed the questionnaires, demographics data is shown in Table 1. The average age of participants surveyed was 50.7 ± 12.5 years of age. There were an approximately equal number of male and female participants (14 males and 12 females). The average wait time at the time of completion of the surveys was 216 with a range of 31–425 days in patients who volunteered to complete the study. Demographics data was compared between the qualitative and questionnaire groups of patients to ensure homogeneity between participants from the two groups. Sex, average age, wait times, and SNOT-22 scores in the qualitative group of patients were similar to that of the questionnaire only group and no statistical difference was found between these two groups (*P* = 0.96 for SNOT-22).

**Table 1:** Patient demographics from the questionnaire group

Characteristic	Average (SD)
All patients	*n* = 26
Age	50.7 (12.5) years
Male	*n* = 14
Previous ESS	12
Wait time^a^	216 (104) days
Median wait time	255 days

^a^As calculated by subtracting the time when the patient completed the questionnaires and when they consented for surgery

### SNOT-22

**Fig. 3:**
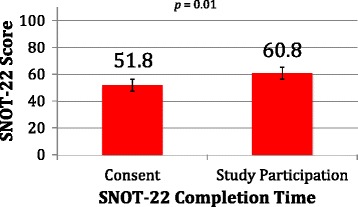
SNOT-22 score at time of consent compared to completion by same participants at the time of the study

The average SNOT-22 score in this group was 60.8 ± 23.9, which is equivalent to scores seen in CRS patients [18]. The four most frequently reported worst symptoms on the SNOT-22 were nasal obstruction, lack of a good night’s sleep, waking up tired, and loss of smell or taste. A comparison was made between the SNOT-22 score from the questionnaires and from a baseline SNOT-22 score done at the time of their consent for surgery. Their scores at the time of consent were an average of 51.8, demonstrating an increase in this cohort by a score of 9 during their present wait, using a paired *t*-test analysis (*p* = 0.01) (Fig. 3).

### HADS outcomes

The HADS tool allows for two-factor (anxiety and depression) analysis within a single clinical measurement tool [19]. The average score for the anxiety scale (HADS-A) was 7.1 ± 4.2 with nine people scoring 8 or higher. The depression scale (HADS-D) average was 7.6 ± 5.2 with thirteen people scoring 8 or higher. The total score average for the HADS was 14.7 ± 8.6. Scores of ≥8 the HADS-A and HADS-D scales suggest a high likelihood of clinically significant anxiety or depression, respectively. A score of >15 for the HADS scale increases the positive predictive value of the scales [15]. Its clinical utility has been evaluated in previous literature and has been shown to have good (specificity 80 %, sensitivity 90 %) validity as a screening measurement of generalized anxiety [20, 19]. No correlation found between the total HADS score and the length of wait experienced by patients (R^2^ < 0.1) in our linear regression analysis.

### WPAI-GH outcomes

According to the questionnaire, 18 of the 26 patients answered that they were working at the time of the study. At the time that the survey was administered, the workers reported absence from their jobs 4.8 ± 13.6 % as a result of their CRS related health issues. The questionnaire findings show that despite a relatively low amount of missed work days, the reported impairment was 34.4 ± 27.9 % with a range between 0 and 90 % as a result of their symptoms. A much greater degree of impairment was found in non-work related activities (i.e., recreational, household, etc.), 50.8 ± 20.5 % with a range between 0 and 90 %. Findings are summarized in Table 2.

**Table 2:** WPAI-GH questionnaire results

Patients who worked for pay	*n* = 18	Mean (SD) %
Percent of work time missed due to health	18	4.8 (13.6)
Percent impairment while working due to health	18	34.4 (27.9)
Percent overall work impairment due to health	18	36.4 (30.1)
Percent activity impairment due to health	26	50.8 (29.5)

## Qualitative interview results

Deductive thematic analysis of the 15 interviews yielded the following recurring themes:

### There is a multifactorial contribution to patients’ frustration with being on a lengthy wait list for surgical treatment for CRS

Patients stated that despite expectations of a wait, it was longer than expected. Other unrelated health care experiences were used in several instances to reflect upon their experiences positively or negatively.

*“I wasn’t surprised because I know that’s how it is in Canada for most surgeries, but I didn’t expect it to be this long for the wait. My friend went into another doctor for her tonsils on the same day I did and she got a call back a month later and was booked in for 3 months later. So I was expecting a shorter wait.”*

*“Within the time I’ve been waiting for [sinus surgery], I’ve been diagnosed, treated, and I’m on the recovery road from a hip replacement surgery…that didn’t take long at all.”*

Fear of progression of CRS and the unpredictability of their symptoms was a source of personal feelings of anxiety, which was exacerbated by a longer wait.

*“I expected to wait because it wasn’t an urgent situation. As I’ve said, I’ve lived with it for decades. If I have to wait a year, I was totally okay with that…If there was a situation where my health was at risk (i.e., cancer), I would be concerned and anxious. But in this case, none.”*

### Having a predetermined date regardless of wait time for surgery was the only thing which was persistently described as something that would improve their experience while being on a wait list for surgery

*“It would be helpful if I had a date for [this surgery], even if it’s in the future at least I can plan on it. I guess other than that, it’s the anxiety of not knowing when the surgery is. If they said it’s a year from now and given me a date, that would have been helpful.”*

### Symptoms did not contribute to patients feeling depressed

Most patients in this cohort did not have difficulty coping with their illness when prompted, nor did they see the need to develop any specific coping strategies to help deal with the wait or their symptoms.

When asked specifically about feeling depressed, patients did not indicate that they felt depressed about their wait list situation specifically.

*“The bad days, I’m not depressed. I’m kind of sad. I have to go to work and sit there for 8 hours, have my nose plugged up and a stuffy forehead kind of experience. Nothing where I don’t feel like waking up, nothing that bad.”*

There was concern from a number of patients regarding whether there would be progression of their symptoms prior to surgery and the unpredictable nature of CRS exacerbations.

### Patients felt that they were significantly affected in their personal lives in social situations and at home by their CRS symptoms

Seven patients described that their symptoms of CRS directly or indirectly affected their social situations. Patients did not generally avoid social situations but many were self-conscious about their symptoms and had to find ways to manage their symptoms and in some occasions avoided social encounters to avoid embarrassment.

*“I’m a little self-conscious about my nose continuing to run, the need to blow [my] nose, that kind of thing. I wouldn’t say avoiding social situations but certainly…bring Kleenex everywhere I go. I’m aware, if I’m in a social situation, I’m constantly conscious of it. I don’t want have a runny nose while I’m talking to somebody (laughs).”*

Two interviewees described an inability to get restful sleep as a factor that had an impact on their home and social activities.

*“I haven’t let it impact my social life. The only thing is, you’re not a 100 % there if you’re kind of tired. Actually, I would say it has because…I go to bed really early because I’m tired. My friend and my husband all complain that I don’t last past 9[pm], whereas before I would be awake until 1 or 2 at night. Because I don’t sleep well, I get tired easily, especially towards the end of the day.”*

Work productivity was impaired mostly from an inability to get a restful sleep. Work absences were only attributable to periods of acute exacerbations in CRS patients interviewed and were worse during certain times of the year.

*“If you don’t sleep well, it affects your productivity and your focus at work. Also, it’s disturbing to my husband. Sometimes, you know, when I wake up at night, he can tell. He says, “I can tell you didn’t sleep well at all.” Sometimes, I’ll have to go to the living room, get some cushions and sleep in a really inclined position.”*

### A majority of patients described no particularly difficulty coping with their CRS in the context of a waitlist for the following reasons: dealing with a benign disease, strength of the physician-patient relationship, relief they were receiving appropriate treatment, and that they were adequately educated about the process and their disease

These were also factors that resulted in patients tolerating a prolonged wait despite being offered a referral by the primary surgeon to other sinus surgeons with shorter wait times. When asked, twelve of the interviewees in our study suggested that a six month wait should be the maximum amount of time to wait for CRS.

The interviewees described that they understood that CRS was not a life-threatening illness and mentioned that they were willing to accept some wait time for ESS in the face of a budget-restricted health care system.

## Discussion

This is the first study to evaluate patient experience while on a wait list for elective surgery for CRS. Although a previous study has shown that CRS symptom severity correlates with a high HADS score at the time of clinical evaluation [21], what happens to these patients is unknown while they are waiting for surgery. Unfortunately, long wait times for ESS has become common as health care expenditures are increasingly restricted. Therefore, the question arose of whether simply waiting on a wait list for surgery independently had any measurable amounts of psychiatric distress.

Interestingly, many patients in our study reported overall less than expected levels of anxiety and depression as measured on HADS despite a high level of symptom severity as measured by the SNOT-22. This may seem contrary to a finding that pre-operative patients at the time of consent considered the potential wait time to be a significant concern before undergoing elective ESS [22]. Our qualitative research indicated that there were a number of factors allowing them to cope with their disease, thus likely limiting psychiatric distress. Notably, an important recurring theme was that patients felt relieved that a plan of treatment was established with their surgeon in the context of a disease that is chronic and will require ongoing medical therapy. The act of being placed on a waitlist may entirely be somewhat beneficial, regardless of wait time. However, it is important to re-emphasize to patients that surgery itself is not curative but the entire goal of ESS is to enable better long term management and control.

The results from the WPAI in our CRS population mirror those from Stankiewicz et al., examining the at the effect of CRS on work productivity [23]. Although there was minimal absenteeism from the workplace (4.8 % time absent from work) as a result of CRS symptoms, our findings demonstrate high levels of impairment with respect to workplace productivity (36.4 %) and non-work or social activities (50.8 %). Although patients were still able to perform necessary activities (i.e., work attendance), it was clear from our qualitative analysis that home lives and ability to interact socially were significantly impaired almost solely by CRS related symptoms.

ESS has now been established to clearly and effectively reduce CRS symptoms while improving quality of life, at least temporarily [22, 24]. Ours is the second study to demonstrate worsening of CRS symptoms (SNOT-22) during the pre-operative wait period. Smith et al. have previously shown both worsening SNOT-22 scores and increased medication usage while waiting for ESS [22]. The mechanism of worsening symptoms was unclear through our qualitative data. However, patients interviewed for our study described that the continued use and the potential for an increasing reliance on medications (particularly oral antibiotics and oral steroids) was an important contributor to their subjective levels of anxiety. Hypothetically, with increasing wait times for ESS, the combination of both progression of CRS symptoms and increasing reliance on systemic medications could push patients to clinically significant levels of psychological distress.

It is also important to note that there was considerable variability with respect to both SNOT-22 and HADS measurements in the tested population. This raises the question of whether there are smaller subsets of patients who are affected to a greater degree and whether these patients are suffering significant psychiatric morbidity from limited access to timely care. Identifying and triaging patients with a quick clinical screening tool for psychiatric distress such as the HADS may be beneficial for patient mental health and their disease from a health care resource point of view. Patients with higher levels of anxiety and depression tend to utilize more health care resources including antibiotics and physician visits [25].

## Conclusions

This study evaluates the functional and psychological impact on patients with CRS and who are currently on a waitlist for ESS. There was no correlation between the length of wait time and the degree of anxiety and depression among patients and overall, no significant levels of psychiatric distress were found. However, our study adds additional evidence that CRS symptoms can worsen while waiting for ESS and this may manifest itself in impairment in both work and social-related activities. With our qualitative analysis, we were better able to understand the patient experience while being on a waitlist for ESS. Overall, patients largely realized they had a benign chronic condition and felt reassured that they were getting appropriate treatment. The physician-patient relationship that was developed was also important in allowing patients to cope with longer wait times. Nonetheless, most patients felt that six months is the appropriate wait time for sinus surgery. There may be a small subset of patients with more psychological distress that may benefit from having surgery sooner.

## Abbreviations

CRS, chronic rhinosinusitis; ESS, endoscopic sinus surgery; HADS, Hospital Anxiety and Depression Scale; SNOT-22, Sinonasal Outcome Test; WPAI-GH, Work Productivity and Activity Impairment-General Health Questionnaire
